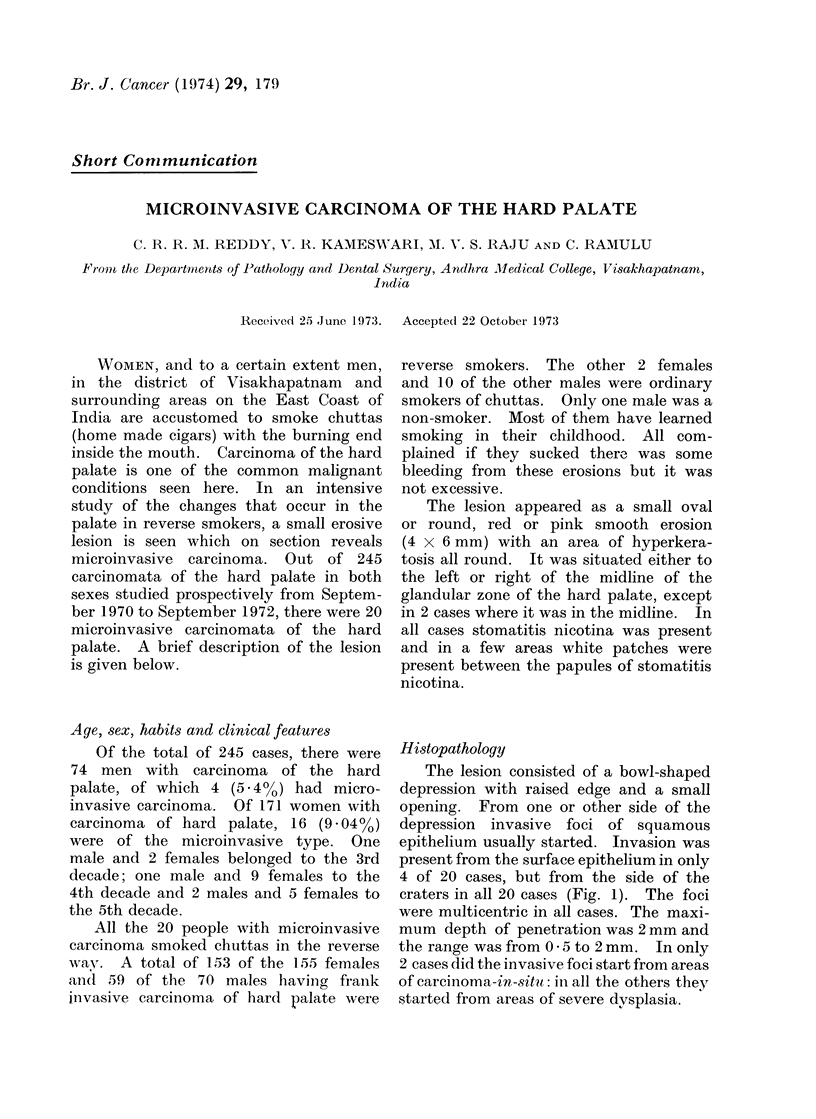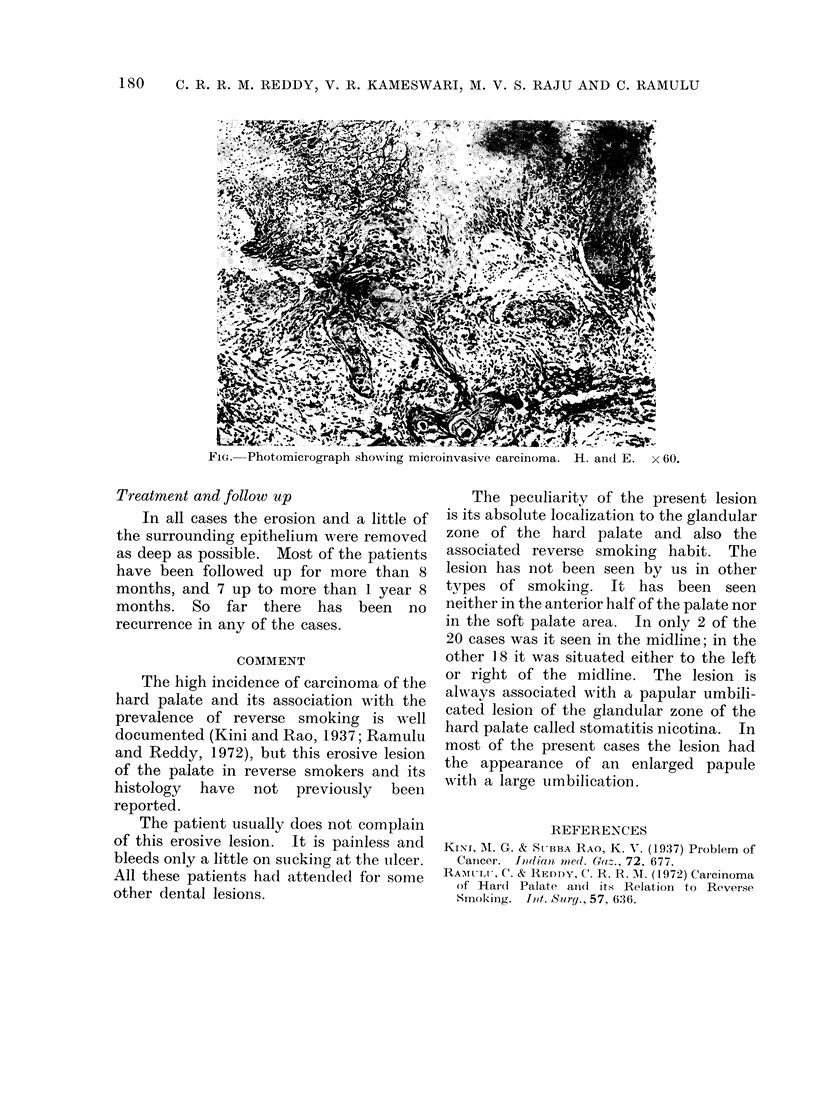# Microinvasive carcinoma of the hard palate.

**DOI:** 10.1038/bjc.1974.55

**Published:** 1974-02

**Authors:** C. R. Reddy, V. R. Kameswari, M. V. Raju, C. Ramulu

## Abstract

**Images:**


*Br. J. Cancer* (1974) **29**, 179

***Short Communication***

### MICROINVASIVE CARCINOMA OF THE HARD PALATE

C. R. R. M. REDDY, V. R. KAMESWARI, M. V. S. RAJU AND C. RAMULU

*From the Departments of Pathology and Dental Surgery, Andhra Medical College, Visakhapatnam, India*

Received 25 June 1973. Accepted 22 October 1973

WOMEN, and to a certain extent men, in the district of Visakhapatnam and surrounding areas on the East Coast of India are accustomed to smoke chuttas (home made cigars) with the burning end inside the mouth. Carcinoma of the hard palate is one of the common malignant conditions seen here. In an intensive study of the changes that occur in the palate in reverse smokers, a small erosive lesion is seen which on section reveals microinvasive carcinoma. Out of 245 carcinomata of the hard palate in both sexes studied prospectively from September 1970 to September 1972, there were 20 microinvasive carcinomata of the hard palate. A brief description of the lesion is given below.

*Age, sex, habits and clinical features*

Of the total of 245 cases, there were 74 men with carcinoma of the hard palate, of which 4 (5·4%) had microinvasive carcinoma. Of 171 women with carcinoma of hard palate, 16 (9·04%) were of the microinvasive type. One male and 2 females belonged to the 3rd decade; one male and 9 females to the 4th decade and 2 males and 5 females to the 5th decade.

All the 20 people with microinvasive carcinoma smoked chuttas in the reverse way. A total of 153 of the 155 females and 59 of the 70 males having frank invasive carcinoma of hard palate were reverse smokers. The other 2 females and 10 of the other males were ordinary smokers of chuttas. Only one male was a non-smoker. Most of them have learned smoking in their childhood. All complained if they sucked there was some bleeding from these erosions but it was not excessive.

The lesion appeared as a small oval or round, red or pink smooth erosion (4 × 6 mm) with an area of hyperkeratosis all round. It was situated either to the left or right of the midline of the glandular zone of the hard palate, except in 2 cases where it was in the midline. In all cases stomatitis nicotina was present and in a few areas white patches were present between the papules of stomatitis nicotina.

*Histopathology*

The lesion consisted of a bowl-shaped depression with raised edge and a small opening. From one or other side of the depression invasive foci of squamous epithelium usually started. Invasion was present from the surface epithelium in only 4 of 20 cases, but from the side of the craters in all 20 cases (Fig. 1). The foci were multicentric in all cases. The maximum depth of penetration was 2 mm and the range was from 0·5 to 2 mm. In only 2 cases did the invasive foci start from areas of carcinoma-*in-situ*: in all the others they started from areas of severe dysplasia.

FIG.—Photomicrograph showing microinvasive carcinoma. H. and E. ×60.

*Treatment and follow up*

In all cases the erosion and a little of the surrounding epithelium were removed as deep as possible. Most of the patients have been followed up for more than 8 months, and 7 up to more than 1 year 8 months. So far there has been no recurrence in any of the cases.

### COMMENT

The high incidence of carcinoma of the hard palate and its association with the prevalence of reverse smoking is well documented (Kini and Rao, 1937; Ramulu and Reddy, 1972), but this erosive lesion of the palate in reverse smokers and its histology have not previously been reported.

The patient usually does not complain of this erosive lesion. It is painless and bleeds only a little on sucking at the ulcer. All these patients had attended for some other dental lesions.

The peculiarity of the present lesion is its absolute localization to the glandular zone of the hard palate and also the associated reverse smoking habit. The lesion has not been seen by us in other types of smoking. It has been seen neither in the anterior half of the palate nor in the soft palate area. In only 2 of the 20 cases was it seen in the midline; in the other 18 it was situated either to the left or right of the midline. The lesion is always associated with a papular umbilicated lesion of the glandular zone of the hard palate called stomatitis nicotina. In most of the present cases the lesion had the appearance of an enlarged papule with a large umbilication.

### REFERENCES

KINI, M. G. & SUBBA RAO, K. V. (1937) Problem of Cancer. *Indian med. Gaz.*, **72**, 677.

RAMULU, C. & REDDY, C. R. R. M. (1972) Carcinoma of Hard Palate and its Relation to Reverse Smoking. *Int. Surg.*, **57**, 636.